# Phenotypic and Marker-Assisted Genetic Enhancement of Parental Lines of Rajalaxmi, an Elite Rice Hybrid

**DOI:** 10.3389/fpls.2016.01005

**Published:** 2016-07-13

**Authors:** Amit K. Dash, Ravi N. Rao, G. J. N. Rao, Ram L. Verma, Jawahar L. Katara, Arup K. Mukherjee, Onkar N. Singh, Torit B. Bagchi

**Affiliations:** Biotechnology Laboratory, Crop Improvement Division, National Rice Research Institute, CuttackIndia

**Keywords:** hybrid rice, MAS, bacterial blight, gene pyramiding, CRMS 32A, CRMS 32B

## Abstract

The cytoplasmic male sterile line system comprising CRMS 32A and its maintainer line CRMS 32B is a popular choice for the development of new hybrids in India as CRMS 32A, having Kalinga 1 cytoplasm (other than WA), is a viable alternative to WA cytoplasm. However, both lines are susceptible to bacterial blight (BB), a major disease on rice. As enhancement of host plant resistance is the most effective and economical strategy to control this disease, four resistance genes (*Xa4, xa5, xa13*, and *Xa21*) were transferred from a BB pyramid line of IR64, into the A and B lines using a marker-assisted backcrossing (MAB) breeding strategy. During the transfer of genes into CRMS 32B, foreground selection was applied using markers associated with the genes, and plants having resistance alleles of the donor, are selected. Selection for morphological and quality traits was practiced to select plants similar to the recurrent parent. The four gene and three gene pyramid lines exhibited high levels of resistance against the BB pathogen when challenged with eight virulent isolates. Using genome wide based SSR markers for background selection, pyramids having >95% of the recurrent parent genome were identified. With CRMS 32B gene pyramid as donor, the four resistance genes were transferred into the A line through repeated backcrosses and the A line pyramids also exhibited high level of resistance against BB. Through a combination of selection at phenotypic and molecular levels, four BB resistance genes were successfully introduced into two parental lines (CRMS 32 B and A) of Rajalaxmi, an elite popular hybrid. The pyramided B lines did exhibit high levels of resistance against BB. Selection for morphological and quality traits and background selection hastened the recovery of the recurrent parent genome in the recombinants. Through repeated backcrosses, all the four resistance genes were transferred to CRMS 32A and test crosses suggest that the maintenance ability of the improved CRMS 32B lines is intact. These improved maintainer and CMS lines can directly be used in hybrid rice breeding and the new hybrids can play an important role in sustainable rice production in India.

## Introduction

Rice is the most important cereal crop in India in terms of area, production and consumption and occupies a prominent place in Indian agriculture. To sustain self-sufficiency and to meet food grain requirement of future, India has to produce 135–140 million tones of rice by 2030. Hybrid rice based on cytoplasmic male sterility (CMS) system has been successfully utilized for improving rice yields by 15–20% over inbred rice varieties (Virmani, 1996). India is the second largest hybrid rice growing country after China. Till date, 78 rice hybrids have been officially released in India and the area under hybrid rice cultivation has reached ~2 million hectares. Like normal inbred rice varieties, hybrid rice is also affected by several biotic stresses (Khush and Jena, 2009). Bacterial blight (BB), caused by Gram-negative proteo bacterium *Xanthomonas oryzae* pv. *oryzae* (*Xoo*), is a devastating disease in the rice growing countries of Asia and causes significant grain yield losses, especially in hybrid rice, worldwide (Chen et al., 2000), because most of the parental lines are susceptible to virulent and prevalent races of *Xoo* under field conditions (Perez et al., 2008). Infection at maximum tillering stage results in blighting of leaves, which eventually causes significant yield losses in severely infected fields ranging from 20 to 30%, but this can reach as high as 80% (Mew et al., 1993; Srinivasan and Gnanamanickam, 2005; Noh et al., 2007). Due to the ineffectiveness of chemicals and antibiotics in controlling the disease, genetic enhancement of host plant resistance through incorporation of major resistance (R) genes is a viable practical option against BB (Khush et al., 1989) as it is the most efficient, economical, and also an environment friendly approach.

In rice, the genetics of resistance to the pathogen has been well characterized. Globally, thirty nine BB resistance genes, series from *Xa1* to *Xa39* have been identified from diverse sources till date and some of those have been characterized (Wang et al., 2014a; Zhang et al., 2014). Of these 39 genes, nine genes, *Xa1, Xa3/Xa26, xa5, xa13, Xa10, Xa21*, *Xa23*, *xa25*, and *Xa27* have been isolated and characterized (Tian et al., 2014; Wang et al., 2014b) and seven genes (*Xa4, Xa7, Xa22, Xa30, Xa31, Xa33*, and *xa34*) have been fine-mapped based on morphological and molecular markers (Kim et al., 2015).

Genetic resistance to rice BB has been extensively used by rice breeders to combat this disease and breakdown of resistance in varieties having a single resistance gene has been reported in rice (Mew et al., 1992; Nelson et al., 1994; George et al., 1997) after 2 or 3 years as a result of shifts in the frequency of pathotypes or the emergence of new ones through mutation or other mechanisms. Multiple resistance genes confer durable broad spectrum resistance through synergistic and complementary gene action to a wide range of races compared to one, two and three gene combinations (Ogawa and Khush, 1988; Huang et al., 1997; Adhikari et al., 1999; Shanti and Shenoy, 2005). A four gene combination (*Xa4*, *xa5*, *xa13*, and *Xa21*) was most stable, conferring resistance against all the isolates of the pathogen (Shanti and Shenoy, 2005; Dokku et al., 2013a,b; Gitishree and Rao, 2015). Breeding parental lines with R genes for resistance to BB has been always part of the breeding program in hybrid rice development (Chen et al., 2001; Borines et al., 2003; Luo et al., 2005; Deng et al., 2006; Zhang et al., 2006; Perez et al., 2008).

Marker assisted selection (MAS) has been proved to be an efficient selection tool for those traits that are difficult and expensive to evaluate in a shorter time period (Tanksley et al., 1989). Marker assisted backcross breeding (MABB) is a breeding strategy where a trait/character is precisely introgressed from a donor parent into the genetic background of a recurrent parent with elite traits using molecular markers and is considered ideal for targeted improvement of elite varieties or parental lines of rice hybrids for one or multiple traits through pyramiding the gene(s) of interest (Hospital, 2005). With the availability of PCR based markers linked to target genes and large number of markers for background selection, pyramiding of these genes is feasible effectively in a minimum number of backcrosses with distinct savings in time, labor, space, and money.

The availability of stable CMS and fertility restoring system is vital for commercial exploitation of heterosis in rice. The cytoplasmic male sterile line CRMS 32A, a highly stable CMS system with full fertility restoration ability, has become a viable alternative to WA, in India, for the development of new hybrids. However, susceptibility to BB of the maintainer line CRMS 32B and CRMS 32A, the cytoplasmic male sterile line, limits their use for their wide application in hybrid rice breeding. Therefore, improvement of BB resistance in CRMS 32B and 32A lines is necessary for their broader use. This study reports the successful improvement of resistance to BB in CRMS 32B and CRMS 32A lines by pyramiding *Xa4*, *xa5*, *xa13*, and *Xa21*, four resistance genes, in these lines, using marker-assisted selection (MAS).

## Materials and Methods

The CRMS 32B, the maintainer line of CRMS 32A, is used as the recurrent (female) parent. The CRMS 32A is a highly stable CMS line having Kalinga 1 cytoplasm, a viable, alternate cytoplasm to the commonly used WA with full fertility restoration (Saidaiah et al., 2010). Till date, two hybrids Rajalaxmi (CRRI, 2009) and KRH 4 (Pushpa et al., 2014) were developed using these parental lines and many promising new hybrid combinations are in active testing stage in both public and private sectors. The CRMS 32B line is of medium duration (90–95 days) with semi dwarf stature (85–90 cm) and long slender grains. But both lines are highly susceptible to BB. The donor parent was CRMAS 2231–2237, an improved line of IR64, incorporated with four genes (*Xa4*+*xa5*+*xa13*+*Xa21*) through MAS and was identified for release for BB endemic areas in India (CRRI, 2008). Conventional backcross approach was followed up to BC_3_ generation and starting from BC_1_F_1_, foreground selection was practiced on >250 plants at each generation for identification of plants with the resistance alleles of the target genes and only the positive plants were advanced to the next generation. Foreground selection was continued till BC_3_F_3_ where pure homozygous plants for all four target genes were identified.

### Screening for Bacterial Blight Resistance

The screening assays against BB were conducted as per Kauffman et al. (1973). Eight virulent isolates of the BB pathogen, *Xanthomonas oryzae* pv. *oryzae* (*Xoo*), collected from Odisha, were used in the study. By screening against these isolates, we could identify resistant lines in our resistance breeding program against BB earlier and these breeding lines/MAS products have demonstrated broad spectrum resistance in the multi-location trials conducted by AICRIP, the nodal agency for validation of results in India. The cultures were prepared by suspending the bacterial mass in sterile water to approx 10^9^ cells/ml. At the maximum tillering stage, three leaves from three different plants of each line were clip-inoculated with two replications. After 21 days of inoculation, observations were recorded and disease score was based on both visual score and lesion length (LL). Plants with an average LL of up to 5 cm were considered resistant and those with LLs above 5 cm were scored as susceptible.

### Characterization for Morphological and Grain Quality Traits

Thirty days old seedlings of the selected lines along with the parents were transplanted in the experimental plots of NRRI at a spacing of 15 cm × 20 cm (within and between rows) with standard agronomic practices. Data was recorded on five plants from each genotype for agronomic traits like plant height, days to 50% flowering, tillers/plant, panicle length, number of filled grains/panicle, and 1,000-grain weight. Characterization of grain and cooking quality traits were performed employing protocols suggested by Ghosh et al. (1971) for Hulling, milling and head rice recovery (HRR), Verghese (1950) for volume expansion ratio (VER), Beachell and Stansel (1963) for water uptake (WU), Little et al. (1958) for alkali spreading value (ASV), Juliano (1971) for amylose content (AC), and Cagampang et al. (1973) for gel consistency (GC).

### Molecular Marker Analysis

The primers employed for the four target BB resistance genes and the Rf genes were all from published reports (**Table 1**). Of the 300 SSRs markers (25 per chromosome) used for parental polymorphism survey, 78 were found to be polymorphic between the parents (range 4–10 per chromosome). These 78 polymorphic markers were used to check the recovery the recurrent parental genome in the gene pyramids. Data was analyzed and similarity matrix was constructed from binary data with dice similarity coefficients and dendrogram was generated with unweighted pair group method arithmatic average (UPGMA) algorithm, using the computer package NTSYS-PC-2.02f (Rohlf, 1998). Graphical Geno Types (GGT) Version 2.0 (Van Berloo, 1999) software program was used for the assessment of the genomic contribution of the parent in the selected recombinants based on SSR data.

**Table 1:** Molecular markers used for amplification of BB and fertility restoration genes.

Gene	Ch No	Linked marker	Reference
*Xa4*	11	MP1, MP2	Ma et al., 1999
*xa5*	5	RG556 (F) RG556 (R)	Yoshimura et al., 1995
*xa13*	8	xa13prom (F) xa13prom (R)	Singh et al., 2011
*Xa21*	11	pTA248 (F) pTA248 (R)	Ronald et al., 1992
*Rf3*	1	RM10305 (F) RM10305 (R)	Shah et al., 2012
*Rf4*	10	RM6100 (F) RM6100 (R)	Singh et al., 2005

### DNA Isolation and PCR Amplification

Mini-scale DNA isolation for PCR analysis was carried out as per Dellaporta et al. (1983). The PCR reaction mixture contained 50 ng templates DNA, 5 pmol of each of the primers, 200 μM dNTPs, 1 unit PCR buffer (10 mM Tris/HCl, pH 8.3, 50 mM KCl, 1.5 mM MgCl_2_, and 0.01 mg gelatin/ml) and 0.5 unit *Taq* DNA polymerase. All the PCR reactions were performed as per earlier reports. The PCR products of CAPS markers RG556 were digested with restriction enzymes *Dra*I as per manufacturer’s instructions. The PCR products and DNA fragments produced by restriction digestions were separated by electrophoresis on agarose gels and analyzed by using Typhoon FLA 700 (Alpha Innotech, USA).

### CMS Line Improvement and Evaluation

To determine their maintaining ability, the improved maintainer lines were test crossed to CMS lines and the F_1_s were evaluated for pollen sterility by collecting spikelets from three panicles of 15 randomly selected plants before anthesis and fixed in 70% ethanol. On a glass slide the pollen grains were mounted, stained with 1% I_2_KI solution and examined under a compound microscope at 40X magnification. The panicles were observed for spikelet sterility. The completely sterile plants, having resistance alleles, were backcrossed to the gene pyramids. At the end of MAS based two back crosses, improved CMS plants having the BB resistance genes in homozygous state were identified. Observations were recorded on the improved CMS lines having three and four genes, for agronomic traits like plant height, DFF, tillers/plant, panicle length, and spikelet sterility (%). The lines were also evaluated for their levels of resistance against BB pathogen.

## Results

### Transfer of BB Resistance Genes

**FIGURE 1:**
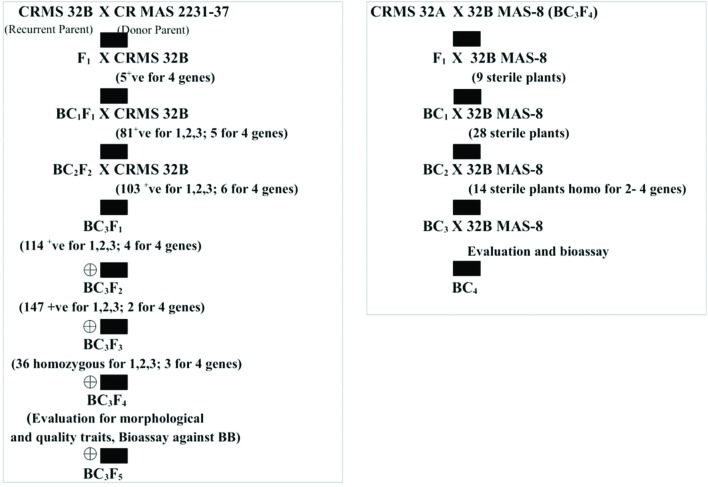
**Flow diagram depicting the different stages of the development of the gene pyramids of CRMS 32B and CRMS 32A**.

The parental polymorphism survey revealed distinct polymorphism for the markers P1 and P2, RG 556, xa13prom, and pTA248 (linked to *Xa4, xa5*, xa13, and *Xa21*, respectively) (**Table 1**) between CRMAS 2231–2237 (resistant) and CRMS 32B (susceptible) suggesting the absence of resistance alleles of all four genes in CRMS 32B (**Figure 1**). In the bioassays conducted against eight isolates of *Xoo*, the donor (CRMAS 2231–2237) showed a high level of resistance (<5 cm of LL), while the recurrent parent showed susceptible reaction with longer LLs (>5 cm).

**FIGURE 2:**
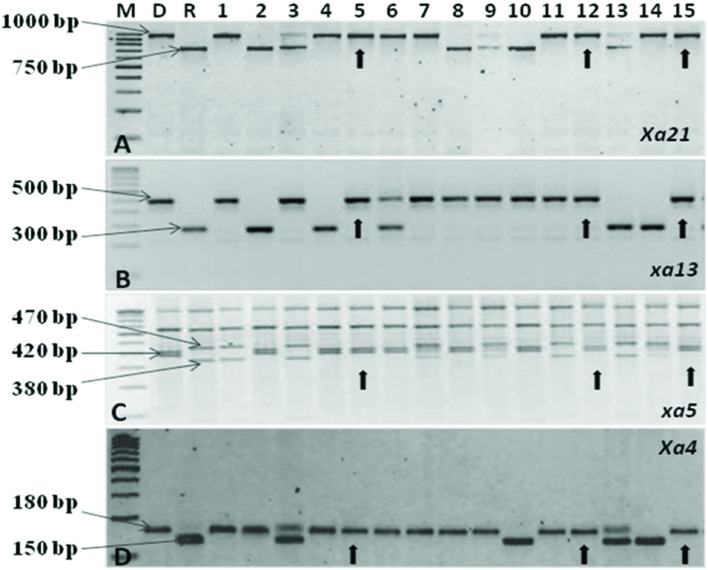
**PCR amplification of resistance genes of bacterial blight. (A)** Amplification of the Xa21 gene using pTA248 primer sequences. **(B)** Amplification of the *xal3* gene using xal3 prom primer sequences. **(C)** Amplification of the *xa5* gene using RG 556 primer sequences followed by digestion of the PCR product with restriction enzyme *Dra1.* **(D)** Amplification of the *Xa4* gene using MP1, MP2 primer sequences M, Marker; D, Donor parent (CRMAS 2231–2237); R, Recurrent parent (CRMS 32B) Nos.1–15 represent sample numbers Arrows indicate a ‘+’ ve plant homozygous for all four genes.

The test cross data on the progeny from the cross CRMS 32A X CRMAS 2231–2237, suggest that CRMAS 2231–2237 is a maintainer for CRMS 32A. The donor parent was tested for its status on The F_1_ plants generated from the CRMS 32B X CRMAS 2231–2237 cross combination were screened for their hybrid status and using the markers and five plants were confirmed to be true F_1_s (**Figure 1**). The backcross breeding was practiced till BC_3_F_1_ generation and at each generation, positive plants for all four resistance genes were selected using marker based foreground selection and very few plants having all four genes were obtained in each generation. The BC_3_F_1_ plants were self-pollinated to produce BC_3_F_2_ and foreground selection was continued to identify plants homozygous for *Xa21*, *xa13*, *xa5*, and *Xa4* genes and selection continued in the BC_3_F_3_ generation also. At each generation >250 plants were evaluated for foreground selection. Interestingly, all 15 possible gene/gene combinations (1 to 4 gene) were found in BC_3_F_3_ generation. three plants were found to be homozygous for all four genes, 13 plants for 3 genes (four different combinations), 15 plants for 2 genes (six different combinations) and presence of only one gene were observed in 8 different plants (**Figure 2**).

### Reaction of Parents and Gene Pyramids against Different Isolates of BB

**FIGURE 3:**
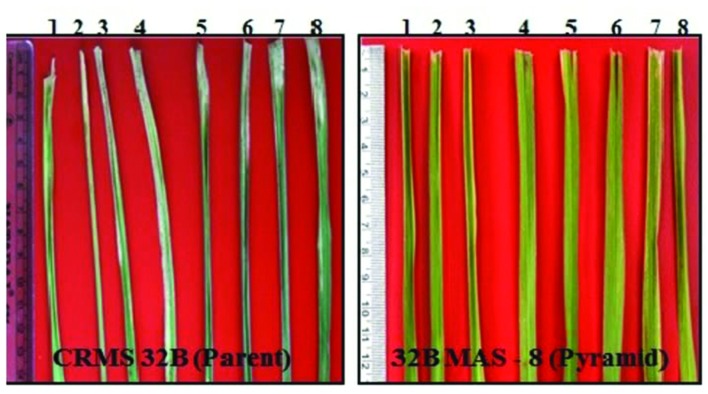
**Reaction of gene pyramids of CRMS 32B against bacterial blight**.

Bioassays conducted against eight different isolates of *Xoo* confirmed the resistance and susceptible reactions of the donor CRMAS 2231–2237 and the recurrent CRMS 32B. CRMS 32B was highly susceptible to all isolates, with LL ranging from 9.0 cm to 16.2 cm, whereas donor line CRMAS 2231–2237 was highly resistant against all isolates, with LL of <0.5 cm (**Table 2**). The pyramid lines having *Xa4* + *xa5* + *xa13* + *Xa21* genes with very small LLs (range 0.7–1.9 cm), showed high levels of resistance to all 8 isolates of *Xoo* (**Figure 3**) while the LLs varied from 1.1 to 3.8 cm in the three gene combination pyramid lines. Though all the gene combinations tested did not show any susceptible reaction to any of the eight isolates employed, the gene pyramids with four genes displayed higher levels of resistance with shorter LLs (<2.0 cm) against all BB isolates while the three gene combinations showed higher levels of resistance against all isolates than lines with two genes.

**Table 2:** Reaction of gene pyramids of CRMS32B against different isolates of bacterial blight (BB).

Line no	Traits	Isolate 1	Isolate 2	Isolate 3	Isolate 4	Isolate 5	Isolate 6	Isolate 7	Isolate 8
CRMAS2231–2237	*Xa21*+*xa13*+*xa5*+*Xa4*	1.1 ± 0.4	1.8 ± 0.7	1.0 ± 0.4	1.6 ± 0.3	0.9 ± 0.2	2.1 ± 0.5	1.6 ± 0.6	0.8 ± 0.3
CRMS 32B	-	10.0 ± 0.5	10.4 ± 0.8	9.0 ± 1.5	9.2 ± 1.3	14.3 ± 1.6	11.1 ± 1.9	10.0 ± 0.6	13.7 ± 0.8
32B MAS-1	*Xa21*+*xa13*+*xa5*+*Xa4*	1.2 ± 0.2	1.5 ± 0.2	0.7 ± 0.3	0.7 ± 0.1	0.8 ± 0.2	1.2 ± 0.2	1.0 ± 0.2	1.2 ± 0.5
32B MAS-4	*Xa21*+*xa13*+*xa5*+*Xa4*	0.7 ± 0.2	0.8 ± 0.4	0.9 ± 0.6	0.7 ± 0.1	1.0 ± 0.3	1.3 ± 0.5	1.5 ± 0.6	0.9 ± 0.5
32B MAS-6	*Xa21*+*xa13*+*Xa4*	3.4 ± 0.4	3.4 ± 0.3	2.9 ± 0.3	3.7 ± 0.6	3.7 ± 0.3	3.4 ± 0.3	3.2 ± 0.7	3.4 ± 0.2
32B MAS-8	*Xa21*+*xa13*+*xa5*+*Xa4*	1.5 ± 0.4	1.9 ± 0.5	1.4 ± 0.5	1.5 ± 0.3	1.3 ± 0.8	1.4 ± 0.3	1.5 ± 0.4	0.9 ± 0.4
32B MAS-9	*Xa21*+*xa13*+*xa5*	2.3 ± 1.9	1.8 ± 1.4	2.1 ± 1.2	2.5 ± 0.5	2.1 ± 1.1	1.3 ± 0.7	2.3 ± 0.9	2.4 ± 1.0
32B MAS-13	*Xa21*+*xa13*+*xa5*	1.8 ± 0.8	2.4 ± 0.7	1.2 ± 0.8	1.7 ± 0.9	1.2 ± 0.4	2.0 ± 0.8	1.1 ± 1.1	2.0 ± 0.9
32B MAS-22	*Xa21*+*xa5*+*Xa4*	2.6 ± 1.4	3.0 ± 0.9	2.1 ± 0.6	2.2 ± 1.2	0.9 ± 0.3	1.4 ± 0.6	2.3 ± 0.9	3.0 ± 1.2
32B MAS-23	*Xa21*+*xa13*+*Xa4*	2.3 ± 0.4	2.8 ± 0.5	3.3 ± 1.1	3.0 ± 0.6	2.7 ± 0.9	3.2 ± 0.9	3.8 ± 1.1	2.4 ± 0.8
32B MAS-29	*Xa21*+*xa5*+*xa4*	3.7 ± 0.6	2.5 ± 0.6	2.6 ± 0.8	3.3 ± 0.5	2.7 ± 0.8	2.6 ± 0.7	2.4 ± 0.8	3.2 ± 0.5
32B MAS-30	*Xa21*+*xa13*+*Xa4*	1.6 ± 0.5	1.9 ± 0.8	2.4 ± 0.6	2.1 ± 0.6	2.2 ± 0.8	3.0 ± 0.7	2.6 ± 0.4	2.4 ± 0.8

### Characterization of Gene Pyramids for Morphological and Grain Quality Traits

At BC_3_F_4_ generation, 43 lines with different gene combinations (1–4 gene) identified and the agronomical and grain quality traits were evaluated in the field and laboratory. The results reveled that most of the traits were virtual identical to those of the recurrent parent, CRMS 32B. Significant differences were not observed for traits like plant height, days to 50% flowering, panicle length, tiller number per plant, fertility % and 1000-grain weight between the recurrent parent and promising gene pyramids and the yield levels are similar (**Table 3**).

**Table 3:** Morphological characteristics of promising gene pyramids of CRMS 32B.

Genotype	DFF	PH	PL	NT	G/P	SF	GW	Y/P	RPG
CRMS 32B	94	87.7	23.4	15	175	86	18.31	15.8	1.00
32B MAS-1	96	82.6	24.2	16	183	84	18.77	16.2	0.84
32B MAS-4	95	84.6	24.2	14	171	79	18.45	15.6	0.83
32B MAS-6	99	92.0	25.6	10	159	77	16.31	15.4	0.92
32B MAS-8	98	87.6	25.6	13	182	88	17.86	15.9	0.95
32B MAS-9	94	89.0	24.0	16	162	80	15.87	16.6	0.84
32B MAS-13	95	88.6	24.8	11	165	85	18.39	15.8	0.93
32B MAS-22	97	88.2	24.0	14	192	85	15.41	15.6	0.93
32B MAS-23	100	92.1	23.2	12	142	74	16.35	15.2	0.88
32B MAS-29	99	86.9	23.2	13	169	80	16.87	15.4	0.86
32B MAS-30	95	85.4	25.8	10	150	75	15.81	15.5	0.89

*DFF, Days to 50% Flowering; PH, Plant Height (cm); PL, Panicle Length (cm); NT, Number of Tillers; G/P, Grains/Panicle; SF, Spikelet Fertility(%); GW, 1,000 Grain Weight (gm); Y/P, Yield/plant; RPG, Recurrent Parent Genome.*

Grain quality analysis was conducted on the grains harvested from ten identified promising lines of BC_3_F_4_ generation that are homozygous for 4 and 3 genes on. The results suggest that all the tested lines were similar to CRMS 32B in quality traits (**Table 4**). All the pyramided lines showed higher HRR value (56.50–64.00) than the parent CRMS 32B (56.00). With respect to ASV, maximum lines are similar to their parental value, i.e., 6. The KLAC values in the lines ranged from 9.1 to 11.5 mm and some are better than CRMS 32B parent (10.4). All the pyramided lines had amylose content in the intermediate range (20.40 to 21.64%) like the parent (22.73 mm).

**Table 4:** Grain quality characters of promising gene pyramids of CRMS 32B.

Genotype	Hull	Mill	HRR	KL	KB	L/B	ASV	VER	KLAC	GC	AMY	WU
CRMS 32B	78.0	65.50	58.00	6.88	2.16	3.19	6	3.75	10.4	35.0	22.73	290
32B MAS-1	80.00	75.50	63.50	7.93	2.63	2.90	6	4.00	11.0	48.0	20.59	255
32B MAS-4	81.00	75.50	64.00	7.30	2.38	3.08	6	4.25	11.6	37.0	20.51	235
32B MAS-6	78.50	70.00	58.50	7.36	2.36	3.13	5	4.00	11.0	32.5	20.40	205
32B MAS-8	80.50	74.00	59.00	6.68	2.32	2.92	6	3.75	10.1	43.5	20.58	240
32B MAS-9	80.00	72.50	61.50	6.69	2.42	2.79	6	4.00	10.8	31.0	20.51	175
32B MAS-13	79.50	74.00	61.50	7.03	2.37	2.99	5	4.25	11.5	31.5	21.15	255
32B MAS-22	79.50	73.50	62.50	7.10	2.59	2.75	5	4.00	11.0	39.0	21.19	250
32B MAS-23	80.50	73.50	61.00	6.71	2.16	3.11	6	3.75	11.0	44.0	21.64	280
32B MAS-29	79.00	73.50	62.00	7.04	2.66	2.68	6	3.75	11.0	48.5	20.48	215
32B MAS-30	79.00	73.50	61.50	6.86	2.44	2.86	6	4.00	10.4	47.5	21.60	305

*Hull, Hulling(%); Mill, Milling(%); HRR, Head Rice Recovery (%); KL, Kernel Length (mm); KB, Kernel Breadth (mm); L/B, Length/Breadth ratio; ASV, Alkali Spreading Value; VER, Volume Expansion Ratio; KLAC, Kernel Length After Cooking (mm); GC, Gel Consistency; AMY, Amylose Content (%); WU, Water Uptake (ml).*

### Genetic Distance and Cluster Analysis

#### Morphological and Grain Quality Traits

**FIGURE 4:**
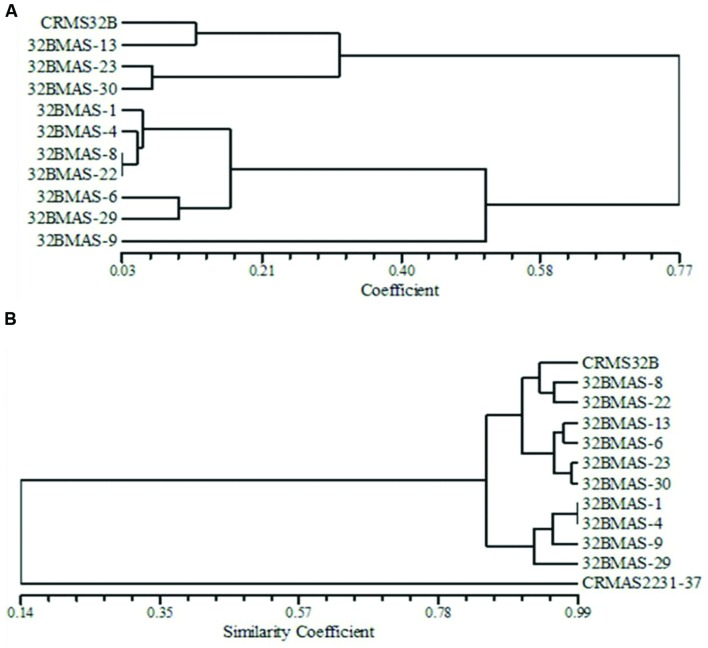
**Dendrograms illustrating the genetic relationship between parents and pyramid lines. (A)** Based on morphological and quality characteristics. **(B)** Based on microsatellite markers.

Based on the dice similarity coefficient dendrogram constructed on morphological and grain quality data, the similarity coefficient varied from 0.03 to 0.77 (**Figure 4A**). All 11 genotypes (10 pyramids and recurrent parent CRMS 32B) were grouped into 2 major clusters. Both the cluster I and cluster II, divided into 2 sub clusters. Cluster I-A consists of 6 lines 32B MAS-1, 32B MAS-4, 32B MAS-6, 32B MAS-8, 32B MAS-22, and 32B MAS-29, while only one line 32B MAS-9 grouped in cluster I-B. Recurrent parent CRMS 32B and line 32B MAS-13 were grouped in cluster II-A, cluster II-B consists of two lines, i.e., 32B MAS-23 and 32B MAS-30.

#### Molecular Data from Microsatellites

Background analysis was carried out with 78 polymorphic SSR markers to assess the recovery of the recurrent parent genome in the selected ten pyramided lines [3 – *Xa4*+*xa5*+*xa13*+*Xa21*; 2 – *Xa4*+*xa5*+*Xa21*; 2 – *xa5*+*xa13*+*Xa21*, and 3 – *Xa4*+*xa5*+*Xa21*]. A total of 162 alleles were observed in the range of 2–4 alleles per marker. PIC values for SSRs ranged from 0.08 to 0.75 with a mean value of 0.59. The similarity co-efficiency among all lines ranged from 0.84 to 0.95 with an average of 0.89 and a high level of genetic similarity between the pyramids and the recurrent parent was observed. A four gene pyramid line, 32B MAS-8, had the highest proportion of the recurrent parent genome (95%) while in three gene pyramids, i.e., 32B MAS-6, 32B MAS-13, and 32B MAS-22, the recovery was 92, 93, and 93%, respectively. In the other six lines, a minimum 89% of the recurrent parent genome was recovered. In the dendrogram generated using the SSR data on the pyramids and the parents, the genotypes were grouped into two major clusters (**Figure 4B**).

The cluster I consists of only the donor parent CRMAS 2231–2237, while cluster II consists of all the pyramided lines and CRMS 32B. Cluster II was further grouped into three sub clusters with II-A having three genotypes (CRMS 32B, 32B MAS-8, and 32B MAS-22), cluster-II-B four genotypes (32B MAS-6, 32B MAS-13, 32B MAS-23, and 32B MAS-30) and cluster II-C having four genotypes (32B MAS-1, 32B MAS-4, 32B MAS-9, and 32B MAS-29).

### Analysis of Genome Introgression on the Carrier and Non-carrier Chromosomes

**FIGURE 5:**
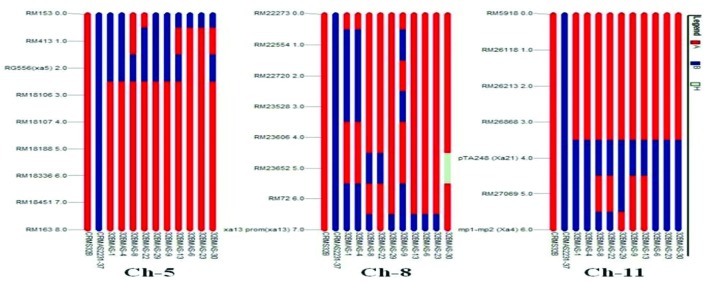
**Graphical genotype analysis of genome introgression associated with BB resistance genes *xa5* (chr 5), *xal3* (chr 8), *Xa4* and *Xa21* (chr 11) in the background of in the gene pyramids of CRMS 32B in BC_3_F_3_ generation**.

The graphical genotyping analysis revealed that in the gene pyramids, the chromosome 1, 2, 4, 6, 7, and 12 of the recurrent parent (CRMS 32B) were completely recovered for 9, 7, 4, 7, 6, and 6 polymorphic marker, respectively. Chromosome 3, 9, and 10 showed introgression of genomic regions from the donor parent CRMAS 2231–2237 even though most of their genomic regions are derived from CRMS 32B. In case of carrier chromosome 5 (*xa5* gene), marker RM413 showed introgression of the donor segment in five lines based on eight marker analysis. Similarly, on the basis of eight polymorphic marker data, three lines showed genetic introgression of the donor segment in chromosome 8 (*xa13* gene). In chromosome 11, out of five polymorphic markers, marker RM27069 present in between *Xa21* and *Xa4* showed introgression of donor segment in six lines while the other four markers showed the recovery of CRMS 32B segments (**Figure 5**).

### Gene Transfer into CRMS 32A and Evaluation of Improved CMS Lines

**FIGURE 6:**
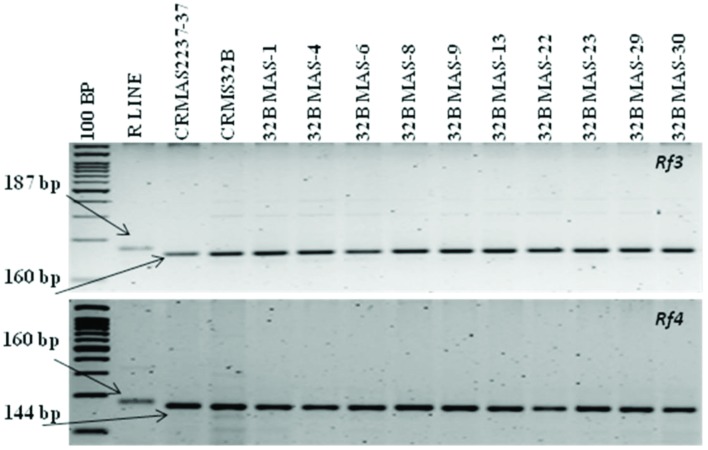
**PCR amplification of fertility restoration genes *(Rf3, Rf4)* in parents and gene pyramids**.

**FIGURE 7:**
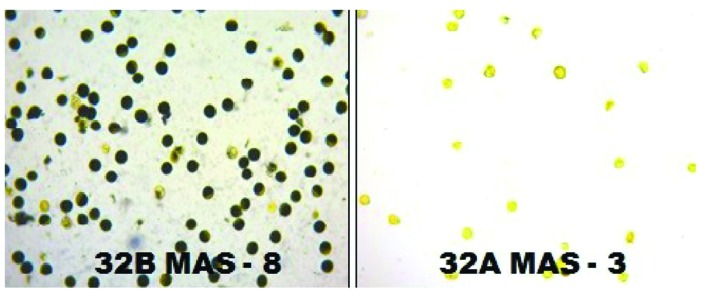
**The fertility and sterility status of the pollen grains in improved lines of CRMS 32B MAS-8 and CRMS 32AMAS-3 (I_2_-KI staining)**.

The molecular analysis conducted for fertility restoration genes (*Rf3* and *Rf4*), the revealed that all the ten selected gene pyramids possess recessive alleles of both the genes like the parents (CRMAS 2231–2237 and CRMS 32B) (**Figure 6**). The test cross results between four highly promising gene pyramids of CRMS 32B (32B MAS-6, 32B MAS-8, 32B MAS-13, and 32B MAS-22) that are similar and CRMS 32A, suggest that the maintaining ability of newly developed gene pyramid B lines is intact as all the F_1_s generated, were completely sterile (pollen as well as spikelet) while the B lines were fully fertile (**Figure 7**). To generate CMS lines homozygous for all four BB resistance genes, two more backcrosses were done using completely sterile plants at each generation and MAS was practiced at each generation (**Figure 1**).

**FIGURE 8:**
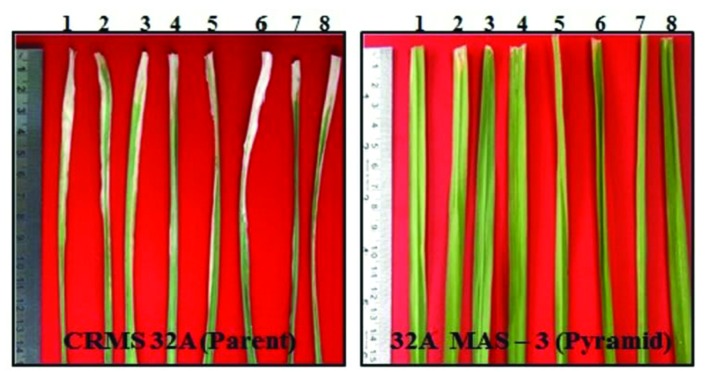
**Reaction of gene pyramids of CRMS 32A against bacterial blight**.

At BC_2_ generation, 14 plants homozygous for 2–4 gene (3 plants having 4 gene, 9 having 3 gene, and 2 having 2 gene) were identified. The program continued with the three gene pyramids and a large number of plants were generated in BC_3_ generation. All the three lines tested for their resistance against BB showed high levels of resistance with lesser LL (0.8 cm to 2.2 cm) while the parental (CRMS 32A) values were in the range of 7.2–12.5 cm (**Figure 8**) With respect to morphological traits, most of the improved lines were similar with CRMS 32A (**Table 5**).

**Table 5:** Morphological characteristics of three gene pyramids of CRMS 32A.

Line no	Gene Combination	DFF	PH	PL	Tiller
CRMS 32A	-	99	82.6	22.6	15
32A MAS-1	*Xa21*+*xa13*+*xa5*+*Xa4*	99	87.4	24.1	16
32A MAS-2	*Xa21*+*xa13*+*xa5*+*Xa4*	97	81.3	21.8	10
32A MAS-3	*Xa21*+*xa13*+*xa5*+*Xa4*	98	88.6	25.6	9

## Discussion

The transfer of BB resistance genes, with effective foreground selection, was achieved in five generations, i.e., three backcrosses followed by two generations of selfing into CRMS 32 B, the maintainer line of Rajalaxmi and the pyramids did exhibit high levels of resistance against BB. Similar results were reported earlier with gene pyramiding against BB (Yoshimura et al., 1995; Huang et al., 1997; Sanchez et al., 2000; Singh et al., 2001; Dokku et al., 2013a,b) indicating the immense potential of the marker assisted approach for genetic enhancement of rice against various stresses.

Parlevliet (1993) suggested that if non-durable major genes are used in physical combination (gene pyramiding) in the same genotype, it may be more difficult for the pathogen to build up races with a wider virulence spectrum and the results of the present study validates such a view as genes in combination were more effective than a single gene. In an earlier study (Dokku et al., 2013b), the breakdown of *Xa4* was observed under Cuttack (eastern India) conditions as lines having only *Xa4* were susceptible to almost all the isolates of BB. Of the gene combinations tested, lines having all four genes expressed higher levels of resistance in comparison to any other combination and the results are in agreement with earlier reports (Yoshimura et al., 1995; Huang et al., 1997; Sanchez et al., 2000; Singh et al., 2001; Dokku et al., 2013a,b; Gitishree and Rao, 2015). The enhanced level of resistance could be the result of synergistic action or quantitative complementation between the resistant genes used (Sanchez et al., 2000). The increased level of resistance conferred by more than one gene governing resistance to a single pathogen race has been described as quantitative complementation (Sanchez et al., 2000). The present results are consistent with Singh et al. (2012) who observed different reactions against different blast isolates in the introgressed lines carrying a single gene (*Piz5* or *Pi54*) in comparison to resistant parents and suggested that broad spectrum resistance against multiple isolates of blast needs pyramiding of several resistance genes.

The objective of the present work was not only to pyramid the resistance genes to enhance resistance against BB but also to keep the genetic base of the parental lines intact as CRMS 32A, with Kalinga 1 cytoplasm, has attracted the attention of hybrid rice breeders as a viable alternative to WA. Two popular hybrids were developed so far using this system and more are in the making. The strategy employed to recover the genetic background of CRMS 32B, after was to employ a combination of morphological (including grain quality) and molecular marker based selections. As compared to the earlier successful reports (Liu et al., 2003; Gopalakrishnan et al., 2008; Sundaram et al., 2008; Basavaraj et al., 2010; Suh et al., 2013; Divya et al., 2014; Balachiranjeevi et al., 2015), we used a higher number of parental polymorphic markers with a better coverage per chromosome for genetic background selection. In our study, a total of 78 polymorphic markers from which 21 from carrier chromosomes (8 marker from chromosome 5 and 11 present *xa5* gene and *xa13* gene, respectively, and 5 markers from chromosome 11 present *Xa21* and *Xa4* gene) were used for background selection as well as to determine the precise parental genomic contribution in carrier chromosomes. This selection strategy was effective as recovery of >90% of the recurrent parent was achieved at BC_3_F_3_ generation. The results on the maintenance ability of the improved lines suggest that all the gene pyramids possess good maintenance ability. Preliminary test crosses with the improved gene pyramids of B with the R line suggest that the heterosis levels remained intact (data not shown).

The strategy appear to be ideal as selection of four plants that are similar to the recurrent parent at BC_3_F_3_ stage suggest that such a strategy has hastened the recovery of the recurrent parent phenotype and the genome in few generations. The graphical genotyping data supports such view as 32B MAS-8 (the line that was used as the donor to transfer genes into A line) had 95% of the recurrent parent genome and it was achieved by following 78 polymorphic markers out of the 300 tested. Since the donor (improved IR 64), and the recipient are improved genotypes, both the genotypes may be sharing several segments of the genome as is evident from the low frequency of polymorphism when tested with 300 markers. The linkage drag problem may be compounded if the donor parent used is a native land race (Stam and Zeven, 1981).

In an earlier study dealing with the improvement of Tapaswini against BB, through MAS (Dokku et al., 2013b), non-recovery of gene combinations like *Xa4+xa5+xa13*, and *Xa4+xa5+Xa21* was observed though the population sizes are adequate (>250) and both the missing combinations involve *xa5* gene specifically. Such selective elimination of gene combinations is not observed in the present study.

The success achieved demonstrates an example of an integrated approach of selection at both molecular and phenotypic levels can achieve both transfer of the desired traits while keeping the recurrent parental genome intact. This approach had advantages like reduction in cost and time and adds precision to the gene transfer programs.

## Conclusion

Development of broad-spectrum resistance against BB in the Indian subcontinent is a major challenge due to the presence of a number of genetically distinct virulent *Xoo* strains in different agro-climatic zones of India. The susceptibility observed in CRMS 32B, the maintainer line and the related CRMS 32A, the cytoplasmic male sterile line against BB has been an impediment to their wide application and the improved versions of the A and B lines can be directly used in the development of new hybrids in India. The study demonstrated that deployment of a four gene combination like *Xa4+xa5+xa13+Xa21* can achieve durable and broad-spectrum resistance against many BB isolates of India. The study clearly establishes the utility of MAS in pyramiding recessive genes like *xa5* and *xa13*, and dominant genes such as *Xa4* and *Xa21* to present a multiple gene barrier against one of the most destructive diseases on rice and provides a platform for the development of hybrid rice varieties with incorporated resistance against BB. The success may also stimulate several such studies to realize the potential of molecular plant breeding as the foundation for crop improvement especially in hybrid development in the 21st century.

## Author Contributions

AD carried out the experiments and data analysis; RR, GR designed the experiments and wrote the manuscript; RV, JK and OS carried out the crossing work and field evaluation; AM carried out bioassays; and TB was involved in conducting quality assays.

## Conflict of Interest Statement

The authors declare that the research was conducted in the absence of any commercial or financial relationships that could be construed as a potential conflict of interest.
